# Estimating Cell Depth from Somatic Mutations

**DOI:** 10.1371/journal.pcbi.1000058

**Published:** 2008-05-09

**Authors:** Adam Wasserstrom, Dan Frumkin, Rivka Adar, Shalev Itzkovitz, Tomer Stern, Shai Kaplan, Gabi Shefer, Irena Shur, Lior Zangi, Yitzhak Reizel, Alon Harmelin, Yuval Dor, Nava Dekel, Yair Reisner, Dafna Benayahu, Eldad Tzahor, Eran Segal, Ehud Shapiro

**Affiliations:** 1 Department of Biological Chemistry, Weizmann Institute of Science, Rehovot, Israel; 2 Department of Computer Science and Applied Mathematics, Weizmann Institute of Science, Rehovot, Israel; 3 Department of Cell and Developmental Biology, Sackler School of Medicine, Tel-Aviv University, Tel-Aviv, Israel; 4 Department of Immunology, Weizmann Institute of Science, Rehovot, Israel; 5 Department of Biological Regulation, Weizmann Institute of Science, Rehovot, Israel; 6 Department of Veterinary Resources, Weizmann Institute of Science, Rehovot, Israel; 7 Department of Cellular Biochemistry and Human Genetics, The Hebrew University-Hadassah Medical School, Jerusalem, Israel; University of California San Diego, United States of America

## Abstract

The depth of a cell of a multicellular organism is the number of cell divisions it underwent since the zygote, and knowing this basic cell property would help address fundamental problems in several areas of biology. At present, the depths of the vast majority of human and mouse cell types are unknown. Here, we show a method for estimating the depth of a cell by analyzing somatic mutations in its microsatellites, and provide to our knowledge for the first time reliable depth estimates for several cells types in mice. According to our estimates, the average depth of oocytes is 29, consistent with previous estimates. The average depth of B cells ranges from 34 to 79, linearly related to the mouse age, suggesting a rate of one cell division per day. In contrast, various types of adult stem cells underwent on average fewer cell divisions, supporting the notion that adult stem cells are relatively quiescent. Our method for depth estimation opens a window for revealing tissue turnover rates in animals, including humans, which has important implications for our knowledge of the body under physiological and pathological conditions.

## Author Summary

All the cells in our body are descendants of a single cell – the fertilized egg. Some cells are relatively close descendants, having undergone a small number of cell divisions, while other cells may be hundreds or even thousands of divisions deep. So far, science was unable to provide even gross estimates for the depths of the vast majority of human and mouse cells. In this study, we show that precise depth estimates of cells can be obtained from the analysis of non-hazardous mutations that spontaneously accumulate during normal development. The concept behind the method is simple: deeper cells tend to acquire more mutations and “drift away” from the original DNA sequence of the fertilized egg. Knowing how deep cells are is the key to many fundamental open questions in biology and medicine, such as whether neurons in our brain can regenerate, or whether new eggs are created in adult females.

## Introduction

Direct observation of cell divisions, which was used to reconstruct the cell lineage of the 959 somatic cells of *Caenorhabditis elegans* [1] implicitly yielded also the depths of these cells. However, direct observations cannot be done for humans and mice since they are opaque and have a tremendous number of cells [2]. Instead, calculations based on cell numbers, proliferation kinetics and various theoretical assumptions have been used to estimate the depths of human [3] and mouse [4] oocytes (approximately 25 cell divisions in both), and of human sperm (approximately 30 divisions at age 15 with additional 23 divisions per year thereafter [3]). The evolutionary-biology concept of a molecular clock [5] – a relatively constant rate of molecular evolution – has also been suggested for estimation of cell depths using either epigenetic [6] or genetic [7] mechanisms. An association between depth and increase in methylation was demonstrated in cell populations of endometrial glands [6]. Mutations in microsatellites (MS; repetitive DNA sequences) have been used to analyze histories of human colorectal tumors, estimating that tumor cells underwent on average about 2,300 divisions since the beginning of tumor progression and 280 divisions since the final clonal expansion [7],[8]. Nevertheless, the depths of the vast majority of human and mouse cells are unknown, and no systematic method for their estimation has been proposed so far.

## Results

### Correlation Between Genetic Distance and Cell Depth

**Figure 1:**
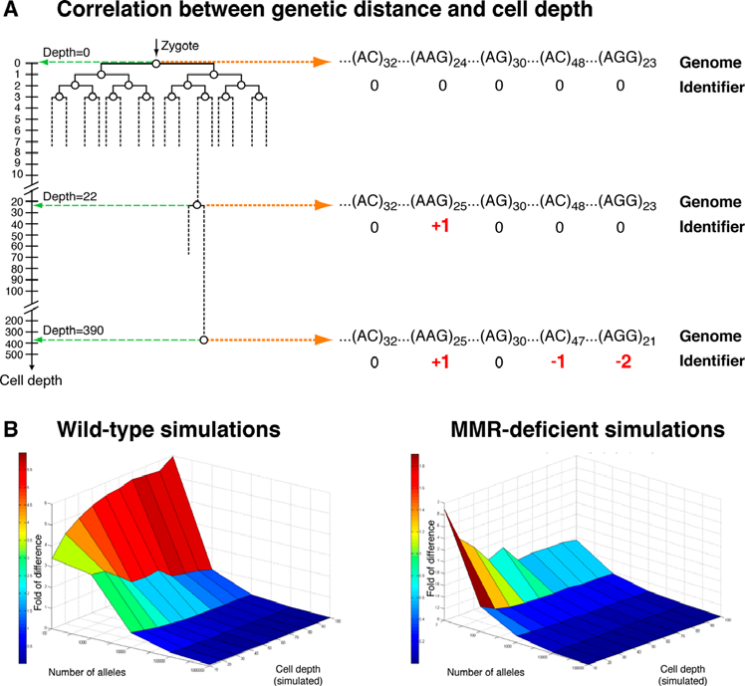
Cell depth analysis. (A) The depth of a cell is the number of divisions it underwent since the zygote. The figure shows a tiny part of the cell lineage tree of an organism – a binary tree representing the exact pattern of cell divisions of its developmental history from a single cell to its current state. The tree depicts not only the lineage relations between cells, but also their depths, obtained by projecting them to the depth axis. A correlation between genetic distance and cell depth is shown in a small fraction (5 MS alleles) of the genome. Each allele is assigned a relative allelic value – a whole number equal to the difference between the number of repeats of that allele and the number of repeat units of the corresponding allele in the zygote. Mutations are coloured in red. (B) Computer simulations of MS mutations and depth estimations based on a maximum likelihood approach. Cells at various depths were simulated accumulating MS stepwise mutations according to wild-type and MMR-deficient mutation rates (*p* = 2.5*10^−5^ and *p* = 0.01, respectively). Depth estimation errors were scored according to the log (base 2) of the ratio between the estimated and simulated depths.

Our work develops the notion of genetic molecular clocks into a quantitative method for depth estimation of single cells of any type. When a cell divides, its DNA is replicated with almost perfect fidelity, yet somatic mutations occur in every cell division [9]. These somatic mutations, which were previously shown to encode the cell lineage tree [9], also encode precise information regarding cell depth. The idea is simple: deeper cells tend to acquire more mutations, hence genetic distance from the zygote and cell depth are strongly correlated (Figure 1A). In principle, any mutation may assist for depth analysis, yet mutations in MS are particularly well suited for depth analysis given that MS are highly abundant in human and mouse [10], and slippage mutations (which insert or delete repeated units) in MS occur at relatively high rates [10] and are coupled to cell division [10]. Moreover, animals with mutations in key mismatch repair (MMR) genes display very high mutation rates in MS [11],[12].

### Computer Simulations

We use the zygote genome as a reference against which mutations are determined (Figure 1A). Each analyzed cell is assigned an identifier [9], a vector representing the mutations that the cell accumulated at a set of analyzed alleles. To assess the theoretical potential of depth analysis using genomic MS we performed computer simulations based on data we previously obtained regarding the numbers of MS and their mutation rates in human and mouse [9]. We simulated wild-type and MMR-deficient mutational behaviour on cells at various depths and estimated their precision using a maximum-likelihood approach (see Materials and Methods). As expected, increasing the number of analyzed alleles, using faster mutation alleles, or analyzing deeper cells – each improves depth estimation score (Figure 1B; a boundary case occurs in wild-type simulations in depth 10 cells, in which error increases with increasing number of alleles, see Materials and Methods). Using the entire set of genomic MS in wild-type human and mouse enables to estimate the depth of cells (at least 10 divisions deep) with precision greater than 95% (Figure 1B). In MMR-deficient organisms, 100 alleles are sufficient to estimate depths of cells (at least 10 divisions deep) with precision greater than 70% (Figure 1B).

### A Method for Estimating Cell Depth from Somatic Mutations

**Figure 2:**
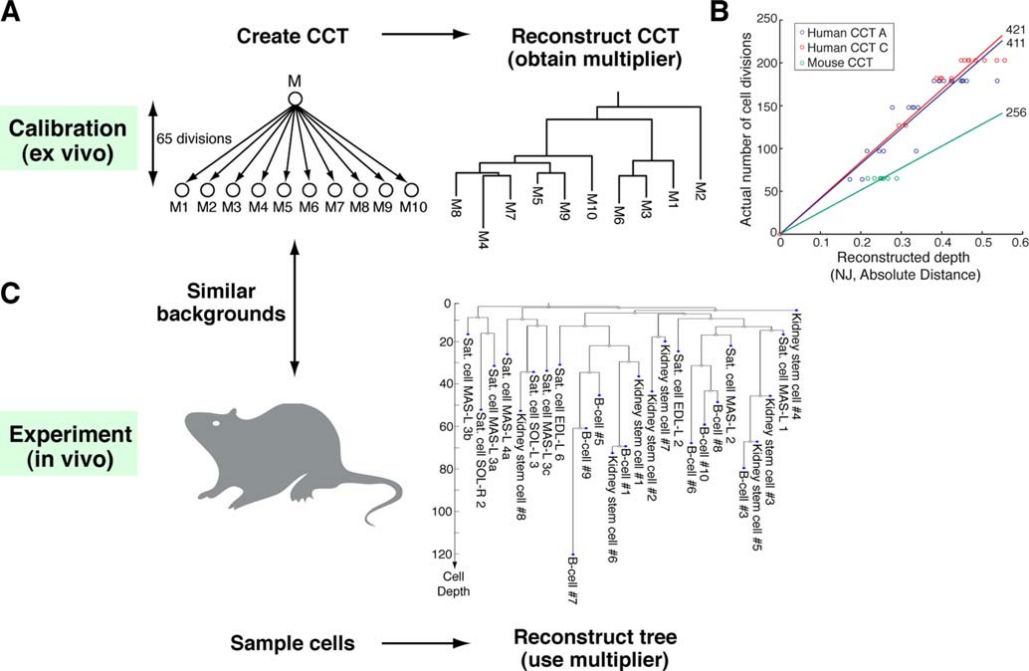
Estimating cell depths from somatic mutations. (A) Our method for *in vivo* cell depth estimation employs a calibration system based on a cultured cell tree (CCT) – an *ex vivo* tree with known topology and well-estimated edge lengths. A CCT created from an Mlh1−/− mouse cell line (of similar background to the mice in which we performed depth analyses) is shown. CCT leaves (M1–M10) were analyzed over a panel of about 100 MS loci, and a tree was reconstructed using the method described in ref. [9] (Neighbor-Joining [NJ] phylogenetic algorithm and ‘Absolute-distance’ distance function were used; see Materials and Methods). Reconstructed depths of all leaves (except for M2, an outlier omitted from analysis) were very similar, with a standard deviation of less than 8% of the mean. (B) Linear correlation between actual and reconstructed depths of human and mouse CCTs. Circles represent CCT nodes; numbers indicate multipliers in each CCT. (C) A multiplier obtained from a CCT is used to calibrate the depths of cells in the reconstructed cell lineage tree.

These simulations assume that the mutational behaviour of MS alleles is simple, consistent, and completely known to us. In practice this is not the case: although some macro-properties of MS mutational behaviour are known [10], the precise behaviour of each MS allele is unknown, and is not readily obtainable. Instead of attempting to decipher the mutational behaviour of every allele in our set of MS, we employed a model-free approach, which utilizes global properties of the set, thus masking the idiosyncrasies of specific alleles. We previously showed that when reconstructing the lineage relations between a set of cells of an *ex vivo* cultured cell tree (CCT) with known depths, there is a linear correlation between the reconstructed and actual depths [9]. Here we continued to investigate this relation, which is the basis of assigning actual depths in our suggested method. A description of the method is shown in Figure 2: a calibration CCT is created and reconstructed using the Neighbor-Joining (NJ) algorithm [13] using the distance function ‘Absolute-distance’ (Figure 2A; this distance measure has previously been suggested to scale linearly with time [14]). A multiplier – a number representing the ratio between the reconstructed and actual depths – is obtained (Figure 2B), and is consequently used for *in vivo* depth estimations (Figure 2C).

### The Method Is Well Supported

Successful depth estimation based on our suggested method depends on the fulfilment of three conditions: (i) there is a good linear correlation between reconstructed and actual node depths in CCTs; (ii) this correlation is similar between similar experiments, i.e. a multiplier obtained from the correlation in one experiment can be used in another; (iii) this multiplier can be reliably transferred from *ex vivo* to *in vivo* experiments. Below we show that each of these conditions is indeed fulfilled. Figure 2B shows a linear correlation in human CCTs [9] (R^2^ = 0.94 and 0.87 for CCTs A and C, respectively) and a newly-created mouse CCT. Multipliers of human CCTs are very similar – 411 and 421 for CCTs A and C, respectively. Depth estimations of nodes from one CCT based on a fit obtained from the other CCT are extremely precise: the average error when estimating the depth of a node from CCT A based on a fit obtained from CCT C is 6.4%±4.1% (and 11%±11% vice versa, for the estimation of CCT C nodes based on a fit obtained from CCT A). The multiplier of mouse CCT is different (256), reflecting the differences between mutational behaviour of our human and mouse MS sets. To further demonstrate that multipliers can be transferred between similar experiments, we performed computer simulations in which a multiplier obtained from one randomly generated tree was used to estimate depths of cells of other similar random trees. These simulations show that when 100 alleles (with mutation rate *p* = 1/100) are analyzed, depths of 90% of the samples are estimated with mean error of less than 30% (data not shown). To show that *ex vivo* and *in vivo* mutation rates are consistent we analyzed the percent of mutations in a set of 130 MS alleles in the mouse CCT samples and multiple samples obtained from four Mlh1−/− mice. The correlation coefficient (r = 0.44) was found to be highly significant (p<10^−6^).

Next we checked whether depth analysis is sensitive to the number of analyzed cells and the specific choice of analyzed alleles. To test the former, we generated random trees with 50 leaves and simulated MS stepwise mutations at various rates. We reconstructed the trees, with increasing subsets of leaves (3–50). Depth estimates of a single leaf (included in all subsets) varied by less than 5% between reconstructions demonstrating that our method is robust to the number of analyzed cells (data not shown). To test the latter, we calculated a fit for the mouse CCT by bootstrapping the data 1000 times (see Materials and Methods), obtaining a mean fit of 251±28. This demonstrates that the obtained multiplier (256) is not sensitive to the specific choice of alleles.

### Depth Estimations of Cells *In Vivo*

**Figure 3:**
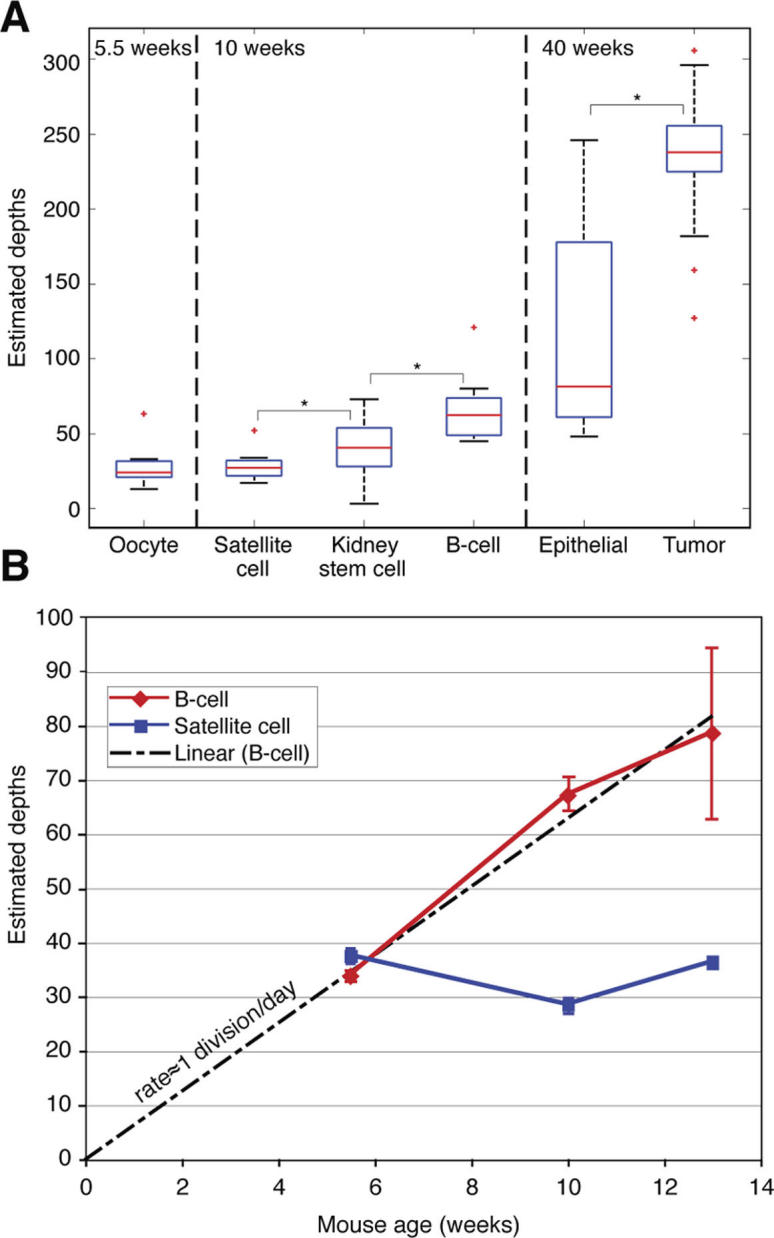
*In vivo* depth estimations. Depth estimates of various cells sampled from mice aged 5.5–40 weeks. (A) Box plots of depths according to cell type and mouse age. Box (blue) displays the middle 50% of the data from the lower to upper quartiles (median is red). Ends of vertical lines (whiskers) indicate minimum and maximum data values, unless outliers (marked by ‘+’) are present, in which case the whiskers extend to a maximum of 1.5 times the inter-quartile range. Stars depict cell types with statistically significant different average depths (p<0.05). (B) Average depths of satellite cells and B cells as a function of mouse age. While depths of satellite cells did not correlate to age, depths of B cells showed a linear correlation (R^2^ = 0.97) to age, corresponding to about one cell division per day. Error bars denote standard errors of the mean.

We applied the method and estimated depths of 163 cells of various types sampled from four MMR-deficient (Mlh1−/−, see [15]) mice aged 5.5–40 weeks (Table 1 and Table S1). Identifiers of analyzed cells (see Table S4) were obtained elsewhere [16],[17] (Text S1 describes materials and methods for obtaining identifiers cells from mice aged 5.5–13 weeks). Each identifier [9] represents the mutations that the corresponding cell sample acquired at the set of loci in comparison to the zygote. Experiment mice have a dual genetic background (C57Bl/6 X 129SvEv), therefore the two alleles of each MS locus are potentially heterozygous, and are considered to mutate completely independent. Our analysis of eight oocytes isolated from the right ovary of a 5.5 week old mouse showed that their average depth is 29 cell divisions (Figure 3A), slightly higher than previous estimates of about 25 cell divisions [4]. The average depth of four types of adult stem cells (satellite, kidney, mesenchymal, hematopoietic) from mice 5.5–13 weeks old ranged from 24–40 cell divisions. These results, when contrasted to the observed depth of differentiated cells such as B-cells (see below), lend support to the view that a general trait of stem cells is their relative quiescence [18]. Satellite cells are adult stem cells positioned under the basal lamina of muscle fibers, which are responsible for the remarkable regenerative capacity of adult skeletal muscle [19]. Depths of satellite cells isolated from various muscles and myofibers ranged from 14 to 75. This wide range of depths suggests that the progenitors of some cells were activated due to events of muscle repair [20] or that satellite cells are a heterogeneous population, for example with respect to the rate of cell division [21]. Nevertheless, the average depths of satellite cells were quite similar (38, 28 and 36; Figure 3B) even though they were sampled from mice at different ages (5.5w, 10w and 13w, respectively). This suggests that most of the satellite cell population in various muscles and myofibers originated during embryonic development, without extensive proliferation in adult life under normal circumstances, and confirms that satellite cells are mitotically quiescent in mature muscle [22]. In contrast, there was a linear correlation between the average depth of B-cells and mouse age (R^2^ = 0.97; Figure 3B). The slope of the linear correlation (6.3) suggests that the turnover rate of splenic B-cells (or progenitors) is about once per day in adults mice. Moreover, B-cells were deeper than satellite cells in 10–13 week old mice (statistically significant in 10 weeks, but not in 13 weeks). Comparison of lung epithelial cells and tumor cells (from various tumor foci) isolated from a 40 week old mouse showed that tumor cells are significantly deeper than epithelial cells (average depths 237 and 117, respectively; Frumkin D., *et al.*, submitted). Two possible explanations for this large difference are that tumor cells divide more rapidly than normal epithelial cells, or that the tumor founder cell was deeper than the epithelial cells, creating a shift for the entire tumor cell population. Another possible explanation is that tumor cells acquire mutations faster than wild type cells. While this may be true in general, it is unlikely in this specific example, since both normal and tumor cells in our Mlh1−/− mice are completely deficient in mismatch repair.

**Table 1:** Estimated cell depths according to cell type and mouse age.

Mouse	Cell Type	Source	Estimated depth
ML7 (75), Age: 5.5 wk	Satellite cell (57)	Various muscles and myofibers	37.5±1.9
	Oocyte** (8)	Right ovary	28.6±5.4
	B cell** (10)	Spleen	33.8±3.8
ML2 (26), Age: 10 wk	Satellite cell (10)	Various muscles and myofibers	28.4±3.2
	Kidney stem cell (8)	Kidney	40.1±7.7
	B cell* (8)	Spleen	67.1±8.7
ML4 (25), Age: 13 wk	Satellite cell (12)	Various muscles and myofibers	36.3±3.6
	Mesenchymal stem cell (5)	Femur/tibia	27.6±7.4
	Hematopoietic stem cell (2)	Femur/tibia	24.0±4.0
	B cell* (5)	Spleen	78.6±35.4
	NK cell* (1)	Spleen	42.0
ML8 (37), Age: 40 wk	Tumor** (23)	Thoracic cavity/lung	237.0±8.4
	Epithelial** (14)	Lung	117.3±18.0

Numbers in parentheses represent the number of cells in each experiment/cell type. Estimated depths represent mean (±standard error of the mean) number of cell divisions for each cell type. Cells were amplified either in culture (unmarked), or by whole genome amplification (^*^GenomePlex, Sigma; ^**^GenomiPhi, GE).

The DNA of analyzed cells was amplified either by *ex vivo* culture or by whole genome amplification (WGA; see Table 1). Although mutations might occur in culture, analysis of cell clones is with high probability identical to analysis had it been performed on the clone's founder cell ([9]; fixation for a mutation during cell culture would be largely undetectable because there are no bottlenecks during the procedure). However, WGA might generate artefact mutations [23] leading to increased depth estimates. Our control experiments show that WGA introduces 0.9–1.2% artefact mutations (in GenomiPhi and GenomePlex, respectively; [16]). We recalculated cell depths assuming these artefact mutation levels in WGA samples, obtaining depths smaller by 18% on average, see Materials and Methods).

## Discussion

In conclusion, we developed a method for estimating depths of cells *in vivo* complementary to our previous method for lineage analysis [9], and applied it to various types of mouse cells. The method is composed of two independent steps: first, relative depths are assigned to sampled cells, which are then transformed to absolute depths using an external calibration system in the form of a CCT. Each CCT is applicable only to the analysis of *in vivo* cells of similar background to those of the CCT. Alternative calibrations are also possible, for example, by the fluorescent labelling of cells followed by analysis of their intensity (which dilutes approximately by half in each cell division [24]). Similar depth estimates (16% difference on average, data not shown) can be obtained independent of tree reconstruction simply by correlating the number of mutations and cell depth. In future, calibration may be discarded altogether if reliable *in vivo* depths estimates can be obtained for a certain group of cells, using any method, which can then be used for internal calibration with other cells.

Horwitz and colleagues also recently developed a method for cell lineage analysis based on somatic mutations in polyguanine repeat DNA sequences [25], very similar to our previous method [9]. They reconstructed a tree of cell samples from a 7-month old wildtype mouse, in which branch lengths correspond to the number of cell divisions. In light of the recent rapid technological advances in high-throughput genomic analysis, we also believe that the direction of this methodology is heading towards the analysis of wild-type organisms, including humans. Nevertheless, at present our preference was to analyze MMR-deficient mice because MS in these mice have elevated mutations rates, which enable analyzing a relatively small number of MS with repeat units of two letters (e.g. ‘AC’) and more. Such MS are preferable over mononucleotide sequences because their PCR stutter patterns are much reduced, making analysis more precise and thus more reliable. Analysis of wildtype organisms based on such MS is not practical at present, as it would require analysis of thousands of MS loci. While not ideal, we believe our analysis may give a lot of useful and reliable data since MMR-deficient humans [11] and mice [12] have been shown to develop normally.

The reconstructed tree obtained by Horwitz and colleagues [25] is unrooted, hence it is not possible to infer the depth of their analyzed cells. Analysis of unrooted trees enables to infer the number of cell divisions between any two samples which is the sum of cell divisions from each sample to their common ancestor (this could be referred to as “depth of cell lineage since X”, X being their common ancestor). In our analysis we infer the identifier of the zygote with high precision (based on tail DNA, see [16]), which enables to reconstruct a rooted tree. Based on this, we can estimate the depth of single cells, and use the term “depth” as shorthand for “depth of cell lineage since the zygote”.

Our depth estimations of oocytes were highly similar to previous reports, providing an independent confirmation for the precision and correctness of our method. Nevertheless, depth estimations may be imprecise to some extent due to various factors, such as the stochastic nature of mutations, differences between *ex vivo* and *in vivo* mutation rates, and different mutation rates between different tissues. In this case of the latter, obtaining tissue-specific mutation rates would enable to perform a compensation step, thus minimizing the error in depth estimations. Beyond the potential of static depth analysis at a specific timepoint, iterative depth analysis at various timepoints can reveal the turnover rates of various tissues under physiological and pathological conditions [26]. For example, depths of a stable tissue which does not turnover is expected to remain constant with time, while depths of non-stable tissues are expected to increase at a rate dependant on the turnover rate. An alternative method for qualitatively obtaining relative cell turnover rates between tissues was previously suggested based on retrospective birth dating of cells [26]. Similarly, analysis of injected stable isotopes was used to determine the turnover rate of blood in mice and rats [27]. Our method enables performing precise depth analysis in a non-invasive fashion, which may shed light on several open fundamental questions, such as whether there is regeneration of neurons [28] and oocytes [29] and the *in vivo* dynamics of stem cells.

## Materials and Methods

### Mouse CCT

Mlh1−/− MEF cells (obtained from Michael Liskay, OHSU) were grown in medium composed of DMEM low glucose (Gibco) supplemented with 10% Fetal Bovine Serum, 1% Non-essential amino acids, and Gentamycin (70 µg/ml). The CCT was created as previously described [9]. Cell division rate was estimated as one division per day according to the frequency of routine plate passages. CCT leaves (M1-M10) were genotyped in our set of MS loci using an automated procedure as previously described [9], except that capillary signal analysis was performed automatically with a new computer algorithm we designed [16]. We reconstructed the CCT with a set of 95 MS loci (see Tables S2 and S3 for list of loci and cell identifiers, respectively) using our previous method for lineage reconstruction [9], except that the distance function was ‘Absolute Distance’. In this function the distance between two samples is the average distance between their allelic values in all alleles which were analyzed in both samples. The multiplier of human and mouse CCTs is the slope of the linear regression between actual and reconstructed depths of CCT nodes. When bootstrapping was performed, alleles from each CCT nodes were sampled with replacement, creating pseudo-identifiers of the same size as the original identifiers. The CCT was reconstructed based on the pseudo-identifiers, and multipliers were calculated.

### Computer Simulations

Identifiers of cells at various depths were simulated based on a symmetric stepwise mutation model, according to which each MS allele mutates with probability *p*, and mutations are either +1 or −1 (each with probability *p*/2). Both wild-type (*p* = 2.5*10^−5^) or MMR-deficient (*p* = 0.01) mutation rates were tested. Depths of simulated cells were estimated using the algorithm described below. Estimation score (fold of difference) was defined as follows: score = |log_2_ (estimated_depth / real_depth)|. Normally, increasing depth improves (lowers) the score. However, in shallow cells with slow mutation rates (e.g. cells 10 divisions deep in wild-type simulations) there are usually no mutations, hence the estimated depth is zero and the score increases with depth since the difference between the estimated and real depths increases.

### Algorithm for Depth Estimation (for Computer Simulations)

This algorithm was used for estimating depths of cells in computer simulations (in the case the mutational behaviour of MS is simple and completely known). We assume that the mutational behaviour of each MS allele is defined by a Probability Vector (PV; p_i_ is the probability of the allele to mutate by *i* MS repeats in each cell division; Σp_i_ = 1). For every MS allele, given its initial value (number of repeats) at the root, fill up a table whose rows are indexed by number of repeats, whose columns are indexed by depth (starting at 1 and ending at the maximum conceivable depth of a leaf), and whose (*i*,*j*) entry gives the probability that at depth *j* its value is exactly *i*. This table can be prepared (in advance and stored) using dynamic programming, column after column, using the PV for the particular MS allele. If there are several alleles with the same PV, then only one table per PV needs to be prepared. Then, given a cell identifier, for every depth *d*, take product of the entries in the tables, where for each MS allele the entry taken is the one in column *d* and the row corresponding to the value of the MS allele in the cell. This product is our estimate of the likelihood that the leaf is at depth *d*. The estimated depth is *d* with maximum likelihood.

### *Ex Vivo* – *In Vivo* Correlation

Only alleles which were successfully amplified and analyzed in at least 20% of mouse CCT and Mlh1−/− samples were analyzed. We generated 10^6^ random permutations of the percent of mutations in the *in vivo* samples, obtaining a correlation coefficient for each permutation. No permutation resulted in a higher correlation coefficient.

### Choice of MS for *In Vivo* Studies

The loci for *in vivo* studies were chosen according to the following criteria: (i) loci with a large number of repeats, in attempt to obtain fast mutating loci. Loci with different mutation rates hold different information, and the best loci for depth analysis are those with the highest mutation rates (unpublished analysis); (ii) loci which are amplified well using our primers yielding high quality signals; (iii) loci whose signal can easily be analyzed (e.g. their two alleles are sufficiently far apart).

### *In Vivo* Depth Estimation

A lineage tree was reconstructed for each Mlh1−/− mouse (using NJ and the ‘Absolute Distance’ function), and for each cell the reconstructed depth was obtained, which is the sum of edge lengths in the reconstructed tree. The estimated depth of each cell was its reconstructed depth multiplied by the multiplier obtained from the mouse CCT. We calculated the 95% confidence interval of the regression coefficient, and used its lower and upper bounds as multipliers for obtaining the 95% confidence interval of the depth estimate of each cell. Depth recalculations assuming WGA artefact mutations were calculated as follows: for each WGA sample *m* randomly chosen mutated alleles (*m* = 0.9% or *m* = 1.2% of analyzed signals for GenomiPhi or GenomePlex WGA samples, respectively) were changed to zero, and depths were recalculated. This was repeated 100 times, and a modified depth (mean depth over repetitions) was obtained for each sample.

## Supporting Information

Text S1 Materials and Methods for obtaining cell identifiers for ML2, ML4 and ML7 cells
(0.05 MB DOC)

Table S1 Estimated depths of each analyzed cell
(0.22 MB DOC)

Table S2 List of microsatellite loci used for analysis of mouse CCT
(0.17 MB DOC)

Table S3 Table of identifiers of mouse CCT nodes
(0.05 MB XLS)

Table S4 Cell identifiers for ML2, ML4, ML7 and ML8 samples
(0.61 MB XLS)
