# Supplementary material for: Estimating Cell Depth from Somatic Mutations
Source: PLoS Comput Biol. 2008 May 9;4(5):e1000058. doi: 10.1371/journal.pcbi.1000058 (PMC2275312; doi:10.1371/journal.pcbi.1000058)
Supplement: Text S1 — Materials and Methods for obtaining cell identifiers for ML2, ML4 and ML7 cells (0.05 MB DOC) [file pcbi.1000058.s001.doc]

**Text S1. Materials and Methods for obtaining cell identifiers for ML2, ML4 and ML7 cells**

**Experiment mice:** Mlh1+/- mice were obtained from Michael Liskay (described in [1]) and were maintained at our institute under C57Bl/6 and 129SvEv (kindly provided by Ari Elson, The Weizmann Institute of Science) backgrounds. Mlh1+/-C57Bl/6 and Mlh1+/- 129SvEv were mated to yield Mlh1-/- progeny of dual background, which were used for experiments. ML2, ML4 and ML7 were 10, 13 and 5.5 weeks old respectively when sacrificed, and were genotyped as Mlh1-/-.

**Isolation and culture of satellite cells:** Satellite cells were isolated and cultured as described in [2]. Briefly, EDL, soleus, gastrocnemius and masseter muscles were digested in 0.2% (w/v) collagenase type I (Sigma-Aldrich) at 37°C. Collagenase was reconstituted in Dulbecco’s Modified Essential Medium (DMEM; high glucose, with L-glutamine, 110 mg/l sodium pyruvate, and pyridoxine hydrochloride; supplemented with 50 U/ml penicillin and 50 mg/ml streptomycin; GIBCO Invitrogen). Following digestion, the muscles were triturated with a wide-bore pipette to release single myofibers. Each single myofiber was transferred to a separate 60 ml dish and then to a tube containing 1ml DMEM. Single myofibers were triturated using a 20G needle mounted onto a 1 ml syringe, to disengage satellite cells. The resulting fiber suspension (in 1 ml DMEM) was then dispensed to 12 Matrigel pre-coated wells within a 24-well plate. Clones were observed every other day.

**Isolation and culture of mesenchymal stem cells (MSCs):** MSCs were isolated and cultured as described in [3,4]. Briefly, femurs were cleaned off the soft tissue and epiphysis to allow bone marrow cells (BMC) collection. The BMC were flushed out with DMEM using syringe with 21G needle. To get Colony forming unit-fibroblast (CFU-F) single cell BMC suspensions were diluted to a concentration of 2.5x10^6^ cells/ml in DMEM supplemented with 10% FCS and plated in 24-wells plates. MSC clones from ML7 samples 104 and 105 were created slightly differently: due to uncertainty of a single cell origin of these clones, a second subcloning step was performed, and cells were allowed to proliferate for additional 4-5 days.

**Isolation and culture of** **kidney stem cells:** Kidney stem cells were isolated and cultured as described in [5].

**Isolation and culture of** **hematopoietic** **stem cells:** Hematopoietic stem cells (SCA-1^+^ Lin^-^) were obtained by crushing tibiae and femurs and extracting cells from the bone marrow (BM). The BM cells suspension were passed through a nylon mesh twice to remove connective tissue and clumps of cells. Subsequently, the cells (20X10^6^) were washed and resuspended at 5ml PBS. BM cell suspensions were maintained on ice throughout the purification procedures. Antibodies used for immunomagnetic positive selection were SCA-1 conjugated to microbeads (SCA-1 multisort kit, Miltenyi Biotec, Bergisch Gladbach, Germany). The following lineage marker antibodies conjugated to microbeads were used: CD45R/B220 for the B lineage, CD4/L3T4 and CD8a/Ly-2 for T cell lineage, and CD11b/Mac-1 for myelomonocytic cells. SCA-1^+^ cells were positively selected using the MACS® magnetic bead system (Miltenyi). The microbeads were removed from the magnetic field, thereby allowing the SCA-1^+^ cells to be collected into the multisort release reagent (Miltenyi). Cytofluorimetric sorting of the isolated SCA-1^+^ cells was carried out by double immunofluorescent staining, using the following directly labeled antibodies (obtained from Pharmingen, San Diego, CA): PE-SCA-1/Ly-6A/E (clone E13-161.7), R-APC-CD45R/B220 (clone RA3-6B2), R-APC-CD4/L3T4 (clone GK 1.5), R-APC-CD8a/Ly-2 (clone 53-6.7) and R-APC-CD11b (clone M1/70). Stained cells were resuspended at 1X10^6^ cells /ml in buffer column at 4◦C (on ice). Cells were then sorted for positive SCA-1-PE cells and lineage negative cells-APC (SCA-1^+^ Lin^-^ cells) into a sterile glass tube containing 500µl/ml FCS sterile using ARIA sorter (BD Biosciences, Mountain View, CA). Immediately after purification, 10^3^ cells per ml SCA-1^+^Lin^-^ cells were suspended in 10 ml of culture medium. Cells were counted and diluted to reach an average of 1 cell/600µl culture medium and replated into 96-well culture plates (200µl/well) to establish single cell clones. Single cell clones were cultured for 7 days in 96-wells plate (Nunc, Roskilde, Denmark) in culture medium made with Iscoves Modified Dulbeccos Medium (IMDM) containing 10% FCS, 2mM L-glutamine, 100U/ml penicillin and 0.1 mg/ml streptomycin.

**Isolation of single cells from a suspension:** Single cells were isolated by suspending a bulk of cells in PBS until a dilution of about 1 cell / 0.5μl was obtained. Then, 0.5μl drops of this dilution were placed at the center of multiple wells of a 96-well (flat bottom) plate. Microscopic observation was used to identify wells with exactly one cell.

**Isolation of B-Cells, NK-Cells:** Spleens of experiment mice were dissected and crushed over a 1μm mesh (A.D. Sinun, Israel) into a Petri dish obtaining a cell suspension. The cell suspension was transferred into a 50ml tube, centrifuged (7 min, 1200 rpm) in a 5702 Eppendorf centrifuge. Cells were resuspended in 1ml PBS and counted. Isolation of specifically desired cells was performed by magnetic sorting using the Miltenyi Biotec magnetic microbeads, MACS columns and MACS separator, according to the manufacturers instructions. CD45R (B220) microbeads were used for B cells, and CD49b (DX5) microbeads were used for NK cells. Single B-cells and NK-cells were obtained as described above.

**Isolation of oocytes:** Ovaries were removed and placed in Leibovitz’s L-15 tissue culture medium (Gibco), supplemented with 5% fetal bovine serum (Biolab, Jerusalem, Israel), penicillin (100 IU/ml) and streptomycin (100 µg/ml, Gibco). When isolated from the ovarian follicles, the oocytes were arrested at the first prophase. To maintain meiotic arrest in fully grown oocytes, the phosphodiesterase inhibitor, isobutylmethylxantine (IBMX) (0.2 mM, Sigma), that prevents cAMP degradation was included in the medium of incubation [6]. The follicles were punctured under a stereoscopic microscope in order to release the cumulus–oocyte complexes that were then placed into acidic L-15 medium (pH 6.0) to obtain cumulus-free oocytes. Each oocyte was placed in a 0.2 mL tube (ABgene) in a volume of 5 μL medium. Oocytes were frozen in liquid nitrogen and kept in -80°C until analyzed.

**Whole Genome Amplification of single cells:** was performed using the GenomePlex Single Cell Whole Genome Amplification Kit (Sigma #WGA4) and the GenomiPhi DNA amplification kit (GE Healthcare, UK). The GenomePlex kit was used according to the manufacturer’s instructions. Prior to the GenomiPhi protocol, cell denaturation was performed as follows: one part (2μl) of lysis solution (400 mM KOH, 100 mM DTT and 10 mM EDTA) was added to 0.5 ml tubes, each containing a single cell in 2μl double distilled water. Cells were lysed for 10 min on ice followed by addition of one part (2μl) of neutralization solution (400 mM HCl, 600 mM Tris HCl, pH 6 – prepared by mixing 4 ml of 1 M HCl and 6 ml of 1 M Tris HCl, pH 7.5). At this point each cell was in 6μl solution (instead of 1μl according to the protocol). The GenomiPhi protocol was carried out according to the manufacturer’s instructions, except that amounts of all the ingredients were multiplied by six.

**Cell identifiers:** Because analysis of multiple loci from single cells requires a minimal amount of DNA (typically ~1μg in our case) we first amplified genomic DNA either by ex vivo culturing of single cells or by WGA (as described above), or a combination of these. DNA was extracted from cell clones using the Wizard SV Genomic DNA Purification System (Promega). All Primers were obtained from Applied Biosystems. Some were part of the ABI PRISM® Mouse Mapping Primers v.1.0, and others were designed by us using Primer3 (<http://frodo.wi.mit.edu/>). In each amplification reaction 4 MS loci were amplified together in 25µl including DNA (25-50ng when extracted from cell clones, or about 1% of WGA products), 0.2µM of each primer, 0.2mM of each dNTP (BIOLINE), and 0.625U of Thermo-Start DNA Polymerase (ABgene). Thermal cycling conditions were: (i) 15’ 95°c, (ii) 35 cycles: 1’ 95°c, 1’ 58°c, 1’ 72°c, (iii) 15’ 72°c. Amplified products from three PCR reactions were combined and run on an ABI prism 3130xl Genetic Analyzer machine (Applied Biosystems). Fragment analysis was performed using the GeneMapper v3.7 software accompanying the machine. We used a programmable laboratory robot (TECAN Genesis) augmented with a PCR machine (Biometra TRobot) to perform the liquid handling for PCR, the PCR itself, and the sample preparation for the capillary machine. Capillary signals were analyzed using an automatic signal analysis program that we designed (Wasserstrom A. *et al*, submitted). Manual override was used in about 1.5% of the signals that were defined by the algorithm as problematic, as well as all signals of samples which were amplified using Genome-Plex, due to their high stutter patterns. Analysis assigns to each MS allele in every sample a relative allelic value - a whole number equal to the difference between the number of repeats of that allele and the number of repeat units of the corresponding allele in the zygote (MS slippage mutations tend to insert or delete repeated units). All relative allelic values together compose the identifier ascribed to each cell.

**References:**

1. Baker SM, Plug AW, Prolla TA, Bronner CE, Harris AC, et al. (1996) Involvement of mouse Mlh1 in DNA mismatch repair and meiotic crossing over. Nat Genet 13: 336-342.

2. Shefer G, Wleklinski-Lee M, Yablonka-Reuveni Z (2004) Skeletal muscle satellite cells can spontaneously enter an alternative mesenchymal pathway. J Cell Sci 117: 5393-5404.

3. Benayahu D, Fried A, Zipori D, Wientroub S (1991) Subpopulations of marrow stromal cells share a variety of osteoblastic markers. Calcif Tissue Int 49: 202-207.

4. Liu Z, Graff E, Benayahu D (2000) Effect of raloxifene-analog (LY 117018-Hcl) on the bone marrow of ovariectomized mice. J Cell Biochem 76: 509-517.

5. Dekel B, Zangi L, Shezen E, Reich-Zeliger S, Eventov-Friedman S, et al. (2006) Isolation and characterization of nontubular sca-1+lin- multipotent stem/progenitor cells from adult mouse kidney. J Am Soc Nephrol 17: 3300-3314.

6. Dekel N (1988) Regulation of oocyte maturation. The role of cAMP. Ann N Y Acad Sci 541: 211-216.
