# Supplementary material for: Estimating Cell Depth from Somatic Mutations
Source: PLoS Comput Biol. 2008 May 9;4(5):e1000058. doi: 10.1371/journal.pcbi.1000058 (PMC2275312; doi:10.1371/journal.pcbi.1000058)
Supplement: Table S1 — Estimated depths of each analyzed cell (0.22 MB DOC) [file pcbi.1000058.s002.doc]

**Supplemental Table S1. Estimated depths of analyzed cells**

| **ML2 (10 weeks, 26 samples)** | | | |
| --- | --- | --- | --- |
| Name | Description | Estimated Depth | 95% Confidence Interval |
| ML2_S37 | Satellite Cell: EDL-L 2 | 25 | 22-27 |
| ML2_S38 | Satellite Cell: SOL-R 2 | 52 | 47-57 |
| ML2_S39 | Satellite Cell: MAS-L 3a | 32 | 28-35 |
| ML2_S40 | Satellite Cell: MAS-L 3b | 17 | 15-18 |
| ML2_S41 | Satellite Cell: MAS-L 3c | 34 | 30-37 |
| ML2_S42 | Satellite Cell: MAS-L 4a | 26 | 24-29 |
| ML2_S43 | Satellite Cell: MAS-L 1 | 17 | 15-18 |
| ML2_S44 | Satellite Cell: MAS-L 2 | 22 | 20-24 |
| ML2_S45 | Satellite Cell: EDL-L 6 | 31 | 28-34 |
| ML2_S46 | Satellite Cell: SOL-L 3 | 28 | 25-31 |
| ML2_S47 | Kidney stem cell #1 | 37 | 33-40 |
| ML2_S48 | Kidney stem cell #2 | 44 | 39-48 |
| ML2_S49 | Kidney stem cell #3 | 36 | 32-39 |
| ML2_S50 | Kidney stem cell #4 | 3 | 3-4 |
| ML2_S51 | Kidney stem cell #5 | 55 | 50-60 |
| ML2_S52 | Kidney stem cell #6 | 73 | 66-80 |
| ML2_S53 | Kidney stem cell #7 | 20 | 18-22 |
| ML2_S54 | Kidney stem cell #8 | 53 | 48-58 |
| ML2_S55 | Splenic B-cell #1 | 66 | 60-73 |
| ML2_S57 | Splenic B-cell #3 | 80 | 72-88 |
| ML2_S59 | Splenic B-cell #5 | 45 | 41-50 |
| ML2_S60 | Splenic B-cell #6 | 68 | 61-75 |
| ML2_S61 | Splenic B-cell #7 | 121 | 109-133 |
| ML2_S62 | Splenic B-cell #8 | 49 | 44-53 |
| ML2_S63 | Splenic B-cell #9 | 49 | 44-54 |
| ML2_S64 | Splenic B-cell #10 | 59 | 53-65 |

| **ML4 (13 weeks, 25 samples)** | | | |
| --- | --- | --- | --- |
| Name | Description | Estimated Depth | 95% Confidence Interval |
| ML4_S1 | Mesenchymal SC Tibia Right #1 | 44 | 40-49 |
| ML4_S2 | Mesenchymal SC Tibia Right #2 | 47 | 42-51 |
| ML4_S3 | Mesenchymal SC Tibia Right #3 | 16 | 14-17 |
| ML4_S4 | Mesenchymal SC Femur Right #1 | 12 | 11-13 |
| ML4_S5 | Mesenchymal SC Femur Right #2 | 19 | 17-21 |
| ML4_S6 | Satellite cell: GAS-L1a | 44 | 40-49 |
| ML4_S7 | Satellite cell: GAS-L1b | 28 | 26-31 |
| ML4_S8 | Satellite cell: GAS-L1c | 22 | 20-25 |
| ML4_S9 | Satellite cell: GAS-L1d | 25 | 22-27 |
| ML4_S10 | Satellite cell: GAS-L1e | 31 | 28-34 |
| ML4_S11 | Satellite cell: GAS-L2a | 32 | 29-35 |
| ML4_S12 | Satellite cell: GAS-L2b | 68 | 62-75 |
| ML4_S13 | Satellite cell: GAS-L2c | 37 | 34-41 |
| ML4_S16 | Satellite cell: EDL-R1a | 29 | 26-32 |
| ML4_S17 | Satellite cell: EDL-R1b | 47 | 42-51 |
| ML4_S18 | Satellite cell: MAS-L1a | 36 | 32-39 |
| ML4_S19 | Satellite cell: MAS-L1b | 36 | 32-39 |
| ML4_S20 | Hematopoietic Stem Cell #1 | 20 | 18-22 |
| ML4_S21 | Hematopoietic Stem Cell #2 | 28 | 25-31 |
| ML4_S22 | B-cell #1 | 67 | 60-73 |
| ML4_S24 | B-cell #3 | 19 | 17-21 |
| ML4_S25 | B-cell #4 | 215 | 194-237 |
| ML4_S28 | B-cell #7 | 28 | 26-31 |
| ML4_S29 | B-cell #8 | 64 | 58-70 |
| ML4_S30 | NK-cell #1 | 42 | 38-46 |

| **ML7 (5.5 weeks, 75 samples)** | | | |
| --- | --- | --- | --- |
| Name | Description | Estimated Depth | 95% Confidence Interval |
| ML7_S1 | Satellite cell: GAS-L1a | 35 | 32-39 |
| ML7_S2 | Satellite cell: GAS-L1b | 43 | 39-47 |
| ML7_S3 | Satellite cell: GAS-L1c | 47 | 43-52 |
| ML7_S4 | Satellite cell: GAS-L2a | 23 | 21-26 |
| ML7_S5 | Satellite cell: GAS-L2b | 33 | 30-36 |
| ML7_S6 | Satellite cell: GAS-L2c | 31 | 28-34 |
| ML7_S7 | Satellite cell: GAS-L2d | 31 | 28-34 |
| ML7_S8 | Satellite cell: GAS-L2e | 45 | 41-49 |
| ML7_S9 | Satellite cell: GAS-L2f | 18 | 17-20 |
| ML7_S10 | Satellite cell: GAS-L3a | 18 | 16-19 |
| ML7_S11 | Satellite cell: GAS-L3b | 18 | 16-19 |
| ML7_S12 | Satellite cell: GAS-L3c | 20 | 18-22 |
| ML7_S13 | Satellite cell: GAS-L3d | 60 | 54-65 |
| ML7_S14 | Satellite cell: GAS-L3e | 54 | 48-59 |
| ML7_S15 | Satellite cell: GAS-L3f | 40 | 36-44 |
| ML7_S16 | Satellite cell: GAS-L4a | 49 | 45-54 |
| ML7_S17 | Satellite cell: GAS-L4b | 72 | 65-79 |
| ML7_S18 | Satellite cell: GAS-L4c | 38 | 34-42 |
| ML7_S19 | Satellite cell: GAS-L4d | 53 | 48-58 |
| ML7_S20 | Satellite cell: GAS-L4e | 32 | 28-35 |
| ML7_S21 | Satellite cell: GAS-L4f | 28 | 25-31 |
| ML7_S22 | Satellite cell: GAS-L4g | 26 | 24-29 |
| ML7_S23 | Satellite cell: GAS-L4h | 32 | 29-35 |
| ML7_S24 | Satellite cell: GAS-R1a | 35 | 32-39 |
| ML7_S25 | Satellite cell: GAS-R1b | 21 | 19-23 |
| ML7_S26 | Satellite cell: GAS-R2a | 45 | 41-50 |
| ML7_S27 | Satellite cell: GAS-R2b | 36 | 33-40 |
| ML7_S28 | Satellite cell: GAS-R2c | 25 | 22-27 |
| ML7_S29 | Satellite cell: GAS-R2d | 51 | 46-56 |
| ML7_S30 | Satellite cell: GAS-R2e | 36 | 33-40 |
| ML7_S31 | Satellite cell: GAS-R2f | 32 | 29-35 |
| ML7_S32 | Satellite cell: GAS-R2g | 24 | 21-26 |
| ML7_S33 | Satellite cell: GAS-R2h | 38 | 35-42 |
| ML7_S34 | Satellite cell: GAS-R3a | 22 | 20-24 |
| ML7_S35 | Satellite cell: GAS-R3b | 26 | 23-28 |
| ML7_S36 | Satellite cell: GAS-R4a | 20 | 18-22 |
| ML7_S37 | Satellite cell: GAS-R4b | 50 | 45-55 |
| ML7_S38 | Satellite cell: GAS-R4c | 37 | 33-40 |
| ML7_S39 | Satellite cell: GAS-R4d | 56 | 50-61 |
| ML7_S40 | Satellite cell: GAS-R4e | 16 | 15-18 |
| ML7_S41 | Satellite cell: GAS-R4f | 45 | 41-50 |
| ML7_S42 | Satellite cell: GAS-R5a | 46 | 42-51 |
| ML7_S43 | Satellite cell: GAS-R5b | 41 | 37-45 |
| ML7_S44 | Satellite cell: GAS-R5c | 75 | 68-83 |
| ML7_S45 | Satellite cell: GAS-R5d | 44 | 40-49 |
| ML7_S46 | Satellite cell: GAS-R5e | 45 | 41-50 |
| ML7_S47 | Satellite cell: EDL-L1 | 41 | 37-45 |
| ML7_S48 | Satellite cell: EDL-L2a | 60 | 54-65 |
| ML7_S49 | Satellite cell: EDL-L2b | 55 | 49-60 |
| ML7_S50 | Satellite cell: EDL-R1a | 37 | 34-41 |
| ML7_S51 | Satellite cell: EDL-R1b | 44 | 39-48 |
| ML7_S52 | Satellite cell: EDL-R1c | 28 | 25-31 |
| ML7_S53 | Satellite cell: EDL-R1d | 28 | 25-31 |
| ML7_S54 | Satellite cell: EDL-R2a | 23 | 21-26 |
| ML7_S55 | Satellite cell: EDL-R2b | 65 | 58-71 |
| ML7_S56 | Satellite cell: EDL-R2c | 14 | 13-16 |
| ML7_S57 | Satellite cell: EDL-R3 | 28 | 25-30 |
| ML7_S121 | Oocyte: R17 | 24 | 22-27 |
| ML7_S122 | Oocyte: R18 | 30 | 27-33 |
| ML7_S123 | Oocyte: R19 | 33 | 30-36 |
| ML7_S124 | Oocyte: R20 | 13 | 12-14 |
| ML7_S125 | Oocyte: R21 | 63 | 57-69 |
| ML7_S126 | Oocyte: R22 | 24 | 22-27 |
| ML7_S127 | Oocyte: R23 | 19 | 18-21 |
| ML7_S128 | Oocyte: R24 | 23 | 21-26 |
| ML7_S129 | Splenic B-Cell #1 | 46 | 42-51 |
| ML7_S130 | Splenic B-Cell #2 | 37 | 33-41 |
| ML7_S131 | Splenic B-Cell #3 | 33 | 30-37 |
| ML7_S132 | Splenic B-Cell #4 | 33 | 30-36 |
| ML7_S133 | Splenic B-Cell #5 | 31 | 28-34 |
| ML7_S134 | Splenic B-Cell #6 | 31 | 28-34 |
| ML7_S135 | Splenic B-Cell #7 | 32 | 29-35 |
| ML7_S136 | Splenic B-Cell #8 | 25 | 23-28 |
| ML7_S137 | Splenic B-Cell #9 | 58 | 52-63 |
| ML7_S138 | Splenic B-Cell #10 | 12 | 11-13 |

| **ML8 (40 weeks, 37 samples)** | | |  |
| --- | --- | --- | --- |
| Name | Description | Estimated Depth | 95% Confidence Interval |
| ML8_1_1 | Tumor cell #1 | 225 | 203-247 |
| ML8_1_2 | Tumor cell #2 | 238 | 214-261 |
| ML8_1_3 | Tumor cell #3 | 219 | 198-241 |
| ML8_1_4 | Tumor cell #4 | 225 | 202-247 |
| ML8_1_10 | Tumor cell #5 | 255 | 230-280 |
| ML8_1_11 | Tumor cell #6 | 238 | 214-261 |
| ML8_1_12 | Tumor cell #7 | 245 | 221-269 |
| ML8_1_13 | Tumor cell #8 | 268 | 241-294 |
| ML8_1_14 | Tumor cell #9 | 235 | 212-259 |
| ML8_1_15 | Tumor cell #10 | 248 | 224-273 |
| ML8_3_1 | Tumor cell #11 | 281 | 253-309 |
| ML8_3_2 | Tumor cell #12 | 234 | 211-257 |
| ML8_3_3 | Tumor cell #13 | 266 | 240-292 |
| ML8_3_4 | Epithelial cell #1 | 178 | 161-196 |
| ML8_3_5 | Epithelial cell #2 | 98 | 88-107 |
| ML8_3_6 | Epithelial cell #3 | 191 | 172-210 |
| ML8_3_7 | Epithelial cell #4 | 61 | 55-67 |
| ML8_3_8 | Epithelial cell #5 | 220 | 198-242 |
| ML8_3_9 | Tumor cell #14 | 256 | 230-281 |
| ML8_3_10 | Epithelial cell #6 | 163 | 147-180 |
| ML8_3_11 | Epithelial cell #7 | 75 | 68-83 |
| ML8_3_12 | Tumor cell #15 | 252 | 227-277 |
| ML8_3_13 | Tumor cell #16 | 127 | 115-140 |
| ML8_3_14 | Tumor cell #17 | 237 | 214-260 |
| ML8_3_22 | Epithelial cell #8 | 246 | 221-270 |
| ML8_3_24 | Tumor cell #18 | 218 | 197-240 |
| ML8_3_25 | Tumor cell #19 | 242 | 218-266 |
| ML8_3_26 | Epithelial cell #9 | 82 | 74-90 |
| ML8_3_27 | Epithelial cell #10 | 48 | 43-52 |
| ML8_3_28 | Tumor cell #20 | 296 | 267-326 |
| ML8_3_30 | Epithelial cell #11 | 60 | 54-66 |
| ML8_7_1 | Tumor cell #21 | 159 | 143-175 |
| ML8_7_2 | Tumor cell #22 | 306 | 275-336 |
| ML8_7_3 | Epithelial cell #12 | 61 | 55-67 |
| ML8_7_4 | Epithelial cell #13 | 81 | 73-89 |
| ML8_7_5 | Epithelial cell #14 | 81 | 73-89 |
| ML8_7_6 | Tumor cell #23 | 182 | 164-201 |

**Abbreviations and Nomenclature of satellite cells:**

SAT = Satellite Cell; EDL = Extensor Digitorum Longus muscle; SOL = Soleus muscle; GAS = Gastrocnemius muscle; MAS = Masseter muscle;

<SAT>-<*muscle*>-<*body-side*><*fiber #*><*satellite cell #*>

For example: SAT-GAS-R4e is the 5^th^ satellite cell from the fourth fiber of the Gastrocnemius muscle (right side)
