# Supplementary material for: Estimating Cell Depth from Somatic Mutations
Source: PLoS Comput Biol. 2008 May 9;4(5):e1000058. doi: 10.1371/journal.pcbi.1000058 (PMC2275312; doi:10.1371/journal.pcbi.1000058)
Supplement: Table S2 — List of microsatellite loci used for analysis of mouse CCT (0.17 MB DOC) [file pcbi.1000058.s003.doc]

**Supplemental Table S2. List of MS Loci used for Mouse CCT**

| **#** | **Locus** | **Repeat** | **Dye** | **Forward Primer** | **Reverse Primer** |
| --- | --- | --- | --- | --- | --- |
| 1 | BARVAZ | GA | FAM | TGGCTGCACAAACAAGATAGGAG | CGAAACGTGCTGTGTCCATTTTG |
| 2 | 2924 | AAAGGG | NED | GGTGGGTCTCTGTGGGTTTGAG | TCATCTCCATTAGCACCTGAGCAC |
| 3 | 0382 | AG | VIC | CATCGTGGAAACTGACCCTTCC | TGTGAAGGCACCAAAATTGAGTTTC |
| 4 | D2MIT66 | AC | PET | GTTGCACAGGCAATCAACC | ATCTATCACTGGGGCTGTGC |
| 5 | D2MIT411 | AC | FAM | ACACTCACAACTACGAGATAAAGCC | AGGTCATTAGGGCTGTCTTCC |
| 6 | D1MIT1001 | AC | NED | TTGTGTGTAGTACAGTGTTGGTGG | TGGTTCCTGACATCAATCTCC |
| 7 | D2MIT100 | AC | VIC | GTGTTCCTAAGGTTGTATTTTGGC | GAAATTTGACAATTGCTAGGTGC |
| 8 | D1MIT426 | AC | PET | CTGCCATCCACTACTTGGTG | CAAATGATACAGTGGAAACCCC |
| 9 | 2621 | AC | FAM | ATGAAAAGATGCAAATTCCAGCAC | AGGCTGCCATACACTCCTCCAG |
| 10 | 3464 | AAG | NED | TTCAGTCTCCTCCCATCTGTGC | CGATGTGTTGTGCATTGGTTCC |
| 11 | 2454 | AGTC | VIC | TTCCCCACATCGCTGTAAATGG | TGGCCTGAGACAAAAGCCTAGC |
| 12 | 1663 | AAGC | PET | GAGTGGTTCCTGATGGGGAGTG | CCCACTTCTTCACAGCAATGTTTG |
| 13 | 2492 | AAAGG | PET | ACCCACATAGAGGCAGGGTGAG | TCACAGACTGAGTTGAAGGAGAAGG |
| 14 | 1053 | AAAG | VIC | AGGCCTATCTTTGCCGCAGAC | GCCTGGCATTGTATCTCAGGTTC |
| 15 | 6231 | AGC | NED | ACTCCCCACAGAGGTCACCAAG | GCTGGCTCTCCTGTAGACATTGG |
| 16 | 2462 | AAAGC | FAM | CCAGAGATACATAGTGAGACCATGACG | CTGATGGTCCTGCTGGCTTTTAG |
| 17 | 1463 | CCTCTTCTT | PET | CATCACCCCAGCTCTTTGAATC | TCCCAGAAATATGTTGAACTTCAGTC |
| 18 | 2941 | AG | NED | GTAGGCCTGCAAAGCAGGAGTG | GGTCTGGGCTAGGGTGGGAAG |
| 19 | D1MIT495 | TC/AC | VIC | CCACCTTGCTCCAAAAGAAA | TCTGAGAGGCTGCCACAATA |
| 20 | 7262 | AAC | PET | CTGAGTTGGCAGGCAAAATGTG | TTGCCTCTCAAGCCTTTGTGTC |
| 21 | 4163 | GCCTCCT | PET | TGGCTGGACTGAGATTCCACAG | CAAACCCTTTAGCAGAGCATGG |
| 22 | H610 | G | PET | CTACAGTAGTAGCATGAGAGGTGGTG | CAAAAGAATTTCTCCTTTTACATTGG |
| 23 | H502 | G | PET | TGTTTCCTTCAGACATTTTATCACAG | ACATCACTTGGAACTGTAAACTCAAC |
| 24 | H341 | G | PET | GCCTAGGACATAAGGATGGTAGATTG | TAGGTTGATATGTGAGTGCAAAGAAAG |
| 25 | M1 | GA | PET | CTGCAGGCAGGAAAAAGCTG | GCCTCCCCCTTTTGAGGTTG |
| 26 | M2 | AC | PET | AGGCCACACCTGAGCTTTTAGC | TCTTCCCAATCACCGATTCACC |
| 27 | M3 | TC | PET | GAAGCCAAGTCAGAAATCCGTTC | AGCACTGCTTGGGCAAATAACC |
| 28 | M4 | AAG | PET | ACGGCGTGCCTTTTCATTTTAC | CTTGTCCCTTGCTGCTCATCTG |
| 29 | M5 | CAG | PET | GGGGCCGAAAAGCTACTTTGAC | GTTCTGGGTCCCTTGGGAATG |
| 30 | M6 | AGA | PET | GGACAAACCAATGTGTTCTTGTGTG | TGAGCAGCATCTCTGGAGAACAG |
| 31 | M7 | AAG | PET | TCCAGCCTTCAGTAGGCACAGG | GGACAACTACCACAAAATTCCAAGG |
| 32 | M8 | TCC | PET | AAGTTGCCCAGAGGGGAATGTC | TTCATGGAAATAAACATGCTTCTGG |
| 33 | M10 | TTTC | PET | AGGACTCCTCGGATTCCAAAGC | GCTGTCGCTCTTGCTCAAACAG |
| 34 | M11 | TTTC | PET | AAACTTTATCAGGAGGAAAGTGAAAGC | GGCCACATCACTTTTGAAGCTG |
| 35 | M13 | CTTT | PET | GGGCATAAATTGTTTGTCGCTTG | GTGTGACTGCTCGCTTCCCATC |
| 36 | M15 | CTAT | PET | ACTTGGAGGAGGACGGTGAGAG | TTTACTTAGTGCTCAGCTTGGAAGG |
| 37 | M16 | AAGC | PET | TTCAGGTAGATACATCAGACCTGTGG | AAGTCTTGGGGGAACAGTCGAG |
| 38 | M17 | AGAA | PET | TCTCATGGATGAACCTATAAACAAAGG | AATTGAAAAGTGTGAGCCCATGC |
| 39 | M18 | CAAAG | PET | AGACCAGGCACCACCAGTCAAG | CGTAAAGAACGCAGATAAAGCTTGC |
| 40 | M19 | TCTCT | PET | TGTGCAGGGAAGACTGGATCTG | TGATCATCTCAAGTGTTTTGTCACG |
| 41 | M20 | GGAGA | PET | ATGCTGGCCAGAGCCTTTCTC | CAGTGATCTCTAGGGAGTGAAACAGAG |
| 42 | 5600 | CCGGAG | PET | CCCAATAACTCTTGGAGCCAGAAC | ACTTGGTGCTCATAGCGAGTTGC |
| 43 | H291 | G | PET | CAAAGGAGACCATCTCAAACAAGC | GCACAAAATGGACATATAAAGAATCAG |

**Primers from ABI PRISM® Mouse Mapping Primers v.1.0:**

| **#** | **Locus (Our name)** | **Repeat** | **Dye** | **Locus (Orig. name)** |  | **#** | **Locus (Our name)** | **Repeat** | **Dye** | **Locus (Orig. name)** |
| --- | --- | --- | --- | --- | --- | --- | --- | --- | --- | --- |
| 44 | ABI1 | AC | FAM | D11MIT143 |  | 74 | ABI60 | AC | NED | D9MIT336 |
| 45 | ABI2 | TG | FAM | D4MIT203 |  | 75 | ABI61 | AC | NED | D5MIT98 |
| 46 | ABI4 | AC | FAM | D15MIT44 |  | 76 | ABI62 | AC | NED | D6MIT284 |
| 47 | ABI6 | AC | FAM | D1MIT64 |  | 77 | ABI63 | TC | NED | D18MIT222 |
| 48 | ABI8 | TG | FAM | D1MIT206 |  | 78 | ABI64 | TG | NED | D14MIT170 |
| 49 | ABI9 | TG | NED | D1MIT102 |  | 79 | ABI65 | TG | VIC | D12MIT182 |
| 50 | ABI10 | TG | NED | D17MIT180 |  | 80 | ABI66 | AC | VIC | D8MIT292 |
| 51 | ABI12 | TG | NED | D5MIT146 |  | 81 | ABI68 | TG | VIC | D13MIT16 |
| 52 | ABI14 | AC | NED | D14MIT60 |  | 82 | ABI71 | TG | VIC | D4MIT18 |
| 53 | ABI16 | AC | NED | D9MIT250 |  | 83 | ABI73 | AC | FAM | D16MIT189 |
| 54 | ABI17 | TG | VIC | D11MIT86 |  | 84 | ABI74 | TG | FAM | D6MIT36 |
| 55 | ABI19 | AC | VIC | D8MIT45 |  | 85 | ABI77 | AC | FAM | D7MIT259 |
| 56 | ABI20 | AC | VIC | D11MIT285 |  | 86 | ABI78 | TG | FAM | D3MIT203 |
| 57 | ABI22 | AC | VIC | D12MIT59 |  | 87 | ABI80 | AC | FAM | D1MIT60 |
| 58 | ABI24 | TG | VIC | D4MIT17 |  | 88 | ABI81 | TG | NED | D9MIT201 |
| 59 | ABI26 | TG | FAM | D2MIT242 |  | 89 | ABI82 | AC | NED | D2MIT1 |
| 60 | ABI30 | GA | FAM | D13MIT19 |  | 90 | ABI85 | TG | NED | D10MIT213 |
| 61 | ABI32 | AC | FAM | D8MIT120 |  | 91 | ABI86 | TG | NED | D4MIT209 |
| 62 | ABI33 | AC | NED | D14MIT126 |  | 92 | ABI87 | AC | NED | D15MIT159 |
| 63 | ABI34 | AC | NED | D10MIT233 |  | 93 | ABI89 | AC | VIC | D1MIT316 |
| 64 | ABI38 | AC | NED | D17MIT122 |  | 94 | ABI90 | AC | VIC | D4MIT348 |
| 65 | ABI40 | TG | NED | D2MIT285 |  | 95 | ABI92 | TG | VIC | D18MIT194 |
| 66 | ABI41 | TG | VIC | D14MIT174 |  | 96 | ABI93 | TG | VIC | D9MIT198 |
| 67 | ABI42 | GA | VIC | D1MIT132 |  | 97 | ABI94 | TG | VIC | D15MIT161 |
| 68 | ABI46 | AC | VIC | D12MIT91 |  | 98 | ABI95 | TC | VIC | D19MIT33 |
| 69 | ABI51 | AC | FAM | D5MIT10 |  | 99 | ABI96 | GA | VIC | D19MIT88 |
| 70 | ABI52 | TG | FAM | D1MIT440 |  | 100 | ABI97 | TG | FAM | D19MIT26 |
| 71 | ABI53 | AC | FAM | D2MIT208 |  |  |  |  |  |  |
| 72 | ABI55 | AC | FAM | D5MIT425 |  |  |  |  |  |  |
| 73 | ABI56 | AC | FAM | D10MIT95 |  |  |  |  |  |  |
